# MiR-129-5p is associated with chronic obstructive pulmonary disease status and alleviates cigarette smoke extract-induced human bronchial epithelial cell injury

**DOI:** 10.18332/tid/221191

**Published:** 2026-06-30

**Authors:** Dan Liu, Jingfan Wang, Ping Zhang, Yonggang Qin, Yunchang Pan

**Affiliations:** 1 Department of Respiratory and Critical Care Medicine, The Fifth Hospital of Wuhan, Wuhan, China; 2 Department of Respiratory and Critical Care Medicine, Hanyang Hospital Affiliated to Wuhan University of Science and Technology, Wuhan, China; 3 Department of Respiratory and Critical Care Medicine, Sanming First Hospital Affiliated to Fujian Medical University, Sanming, China

**Keywords:** chronic obstructive pulmonary disease, miR-129-5p, ICAM1, inflammatory

## Abstract

**INTRODUCTION:**

Chronic obstructive pulmonary disease (COPD) is a common progressive respiratory disease with persistent airflow limitation, chronic airway inflammation, and limited early diagnostic biomarkers and targeted therapies. MicroRNAs (miRNAs) are involved in COPD pathogenesis, but the role of microRNA-129-5p (miR-129-5p) in COPD is unclear. This study investigated miR-129-5p’s association with COPD susceptibility, its regulation on cigarette smoke extract (CSE)-induced bronchial epithelial cell injury, and the mechanism involving intercellular cell adhesion molecule 1 (ICAM1) and RELA.

**METHODS:**

The study included a cross-sectional study and in vitro experiments. The cross-sectional study (Hanyang Hospital, March 2022–May 2024) collected serum from 126 COPD patients and 120 controls to detect miR-129-5p, ICAM1, and RELA via quantitative real-time polymerase chain reaction (qRT-PCR). Receiver operating characteristic (ROC) curve area (AUC), multivariate logistic regression, and Pearson correlation were used for analysis. In vitro, human bronchial epithelial cells (BEAS-2B) were divided into 6 groups for the CSE-induced injury model; Cell Counting Kit-8 (CCK-8), flow cytometry, enzyme-linked immunosorbent assay (ELISA), and dual-luciferase assay detected cell functions and miR-129-5p-ICAM1 targeting.

**RESULTS:**

Serum miR-129-5p was significantly downregulated in COPD patients, with elevated ICAM1, RELA, and inflammatory factors. It distinguished COPD from controls (AUC=0.88; 95% CI: 0.84–0.92, p<0.001) and was an independent susceptibility factor (adjusted odds ratio, AOR=0.15; 95% CI: 0.08–0.26, p<0.001). miR-129-5p was negatively associated with inflammatory factors and positively associated with pulmonary function. CSE inhibited miR-129-5p and induced injury; its overexpression reversed injury, knockdown exacerbated it, and it targeted ICAM1, whose overexpression abrogated miR-129-5p’s protection.

**CONCLUSIONS:**

Serum miR-129-5p was downregulated in COPD patients, while cellular evidence indicated that it potentially alleviates CSE-induced injury by targeting ICAM1 and downregulating RELA, providing preliminary evidence for COPD pathogenesis; further large-scale studies are needed to verify its clinical value.

## INTRODUCTION

Chronic obstructive pulmonary disease (COPD) is a common respiratory disorder characterized by persistent, incompletely reversible airflow limitation, accompanied by progressive lung injury and chronic airway inflammation^1,2^. Patients often present with dyspnea, chronic cough, and expectoration, with progressive symptom deterioration that severely impairs quality of life^3,4^. Its incidence remains high and is expected to continue rising over the next decade, with the high morbidity and mortality attracting widespread attention^4^. It is well established that COPD is associated with inflammatory responses mediated by inflammatory factors such as tumor necrosis factor-α (TNF-α) and interleukin-8 (IL-8)^5,6^, but the specific pathogenesis requires further investigation.

MicroRNAs (miRNAs) are key non-coding RNAs that regulate gene expression^7^. Dysregulation of their expression is closely associated with various diseases, and due to their convenient detection in blood and high stability, they have been recognized as novel potential biomarkers for COPD^8-11^. Previous studies have confirmed that multiple miRNAs play pathophysiological roles in COPD: for example, miR-145 can inhibit the release of pro-inflammatory factors from airway smooth muscle cells^12^, miR-483-5p exerts a protective effect in patients^13^, and miR-150-5p has also been shown to be involved in pulmonary inflammation and injury^14,15^.

miR-129-5p is a miRNA with well-documented anti-inflammatory activity, which participates in disease progression by regulating autophagy and inflammatory responses^16^. Existing studies have demonstrated that it can alleviate lipopolysaccharide (LPS)-induced acute kidney injury^17^ and its downregulation is associated with various diseases, particularly common in cancers^18^. For instance, in gastric cancer, it is involved in cancer cell migration and invasion by targeting IL-8^19^ and its expression is significantly reduced in primary lung cancer tissues and cell lines^20^. In view of the anti-inflammatory properties of miR-129-5p and its association with pulmonary diseases, while chronic inflammation is the core pathological feature of COPD, its regulatory role and molecular mechanism in COPD remain to be clarified. Therefore, this study selected miR-129-5p as the research object, aiming to clarify its expression characteristics in the serum of COPD patients. Through clinical sample analysis and in vitro functional experiments, we explored the association between miR-129-5p and COPD, its regulatory effect on the viability and apoptosis of bronchial epithelial cells, and the underlying molecular mechanism of inflammatory regulation, to provide an experimental basis for the study of the pathogenesis of COPD.

## METHODS

This study consisted of two independent but logically linked parts: 1) a single-center cross-sectional observational study to explore the association between serum miR-129-5p expression and COPD; 2) an *in vitro* cell functional experiment to verify the regulatory effect of miR-129-5p on CSE-induced human bronchial epithelial cell injury and its underlying molecular mechanism. The clinical study was conducted in strict accordance with the Declaration of Helsinki and was approved by the Clinical Ethics Committee of Hanyang Hospital affiliated to Wuhan University of Science and Technology (Approval No. 2022-012, 1 March 2022). All participants signed written informed consent forms.

### Study subjects and sample collection

This cross-sectional study was conducted at the Outpatient Department of Respiratory and Critical Care Medicine, Hanyang Hospital affiliated to Wuhan University of Science and Technology, between March 2022 and May 2024. A total of 126 patients with COPD who met the inclusion criteria were enrolled as the COPD group, and 120 age- and gender-matched healthy individuals from the hospital’s Physical Examination Center during the same period were enrolled as the control group.

Inclusion criteria for the COPD group were that they met the diagnostic criteria for COPD in the Global Initiative for Chronic Obstructive Lung Disease (GOLD) 2020 guidelines: post-bronchodilator forced expiratory volume in 1 second/forced vital capacity (FEV_1_/FVC) <70%; aged ≥40 years; complete clinical data, pulmonary function test reports, and qualified serum samples available.

Exclusion criteria for the COPD group were that they had concurrent other chronic respiratory diseases (e.g. bronchial asthma, interstitial lung disease, pulmonary fibrosis, active tuberculosis, lung cancer); complicated with severe cardiovascular diseases, decompensated liver and kidney dysfunction, malignant tumors, active autoimmune diseases, or acute infectious diseases within 4 weeks prior to enrollment; pregnant or lactating women; received systemic glucocorticoid or immunosuppressant treatment within 3 months before enrollment [patients receiving inhaled corticosteroid (ICS) maintenance therapy were not excluded, with detailed medication history recorded].

Inclusion criteria for the control group: aged ≥40 years; no history of chronic respiratory diseases, with normal chest imaging and pulmonary function test results; frequency-matched with the COPD group in terms of age and gender distribution; and no family history of COPD or other hereditary respiratory diseases.

Fasting venous blood samples (5 mL) were collected from all participants within 24 hours of enrollment. The blood samples were centrifuged at 3000 rpm for 10 min at 4^o^C to separate the serum, which was then subpackaged and stored at -80^o^C for subsequent RNA extraction and detection.

### Variable definition and data collection

The following variables were collected from all participants, with clear definitions and data sources as follows.

*Demographic variables*

Gender (male, female), age, and body mass index (BMI, kg/m²) data were collected from the hospital’s electronic medical record system and physical examination reports.

*Smoking status*

Dichotomized into current smoker or non-smoker, with smoking rate calculated as the proportion of current smokers in each group; data were collected via standardized questionnaire survey.

*Pulmonary function indices*

FEV_1_ (% predicted) and FEV_1_/FVC (%) data were collected from standardized pulmonary function test reports, tested by the same professional technician using the same pulmonary function instrument.

*Inflammatory factors*

Serum TNF-α and IL-6 levels (pg/mL) data were obtained from subsequent ELISA detection.

*COPD severity grading*

Graded COPD severity according to the GOLD 2020 guidelines into Grade I (mild), Grade II (moderate), and Grade III (severe)^21,22^; data were collected from the patients’ medical records.

### qRT-PCR

Total RNA was extracted from the serum samples using TRIzol reagent (Invitrogen, USA), and the concentration and purity of RNA were detected by a NanoDrop 2000 spectrophotometer. Complementary DNA (cDNA) was synthesized using the PrimeScript™ RT Kit (with gDNA Eraser, TAKARA, Japan) according to the manufacturer’s instructions. qRT-PCR was performed on the Applied Biosystems 7500 Real-Time PCR System using the SYBR Premix Ex Taq Kit (TAKARA, Japan). U6 was used as the internal reference gene for miR-129-5p, and glyceraldehyde-3-phosphate dehydrogenase (GAPDH) was used as the internal reference gene for ICAM1 and RELA. The relative expression levels of target genes were calculated using the 2^-ΔΔCt^ method. The primer sequences used in this study are shown in Supplementary file Table 1.

### ELISA

The levels of TNF-α and IL-6 in the serum samples were measured using commercial ELISA kits (Elabscience Biotechnology Co., Ltd., Wuhan, China), strictly following the manufacturer’s instructions. The absorbance at 450 nm was detected by a microplate reader, and the concentrations of the target factors were calculated according to the standard curve.

### *In vitro* cell functional experiment

In the second step of this research, we established a CSE-induced human bronchial epithelial cell injury model *in vitro* to verify the regulatory effect and mechanism of miR-129-5p on airway epithelial cell injury and to clarify the biological significance of the clinical findings.

### Cell culture and reagents

Human bronchial epithelial cells (BEAS-2B) and human embryonic kidney 293T (HEK293T) cells were purchased from the Cell Bank of the Chinese Academy of Sciences (Shanghai, China). BEAS-2B cells were cultured in Bronchial Epithelial Cell Growth Medium (BEGM, Lonza, USA, Cat No. CC-3170) supplemented with BEGM BulletKit additives, in a constant-temperature incubator at 37^o^C with 5% CO_2_ and saturated humidity (replacing serum-containing DMEM, which induces epithelial-mesenchymal transition of BEAS-2B cells). HEK293T cells were cultured in DMEM complete medium (Gibco, USA) containing 10% fetal bovine serum (FBS, Gibco, USA, Cat No. 10099141), 100 U/mL penicillin, and 100 μg/mL streptomycin. The medium was changed every 24 h, and cells were passaged when the confluence reached 80–90%. BEAS-2B cells at passage 3–8 were used for all experiments to ensure a stable cell phenotype.

miR-129-5p mimic, miR-129-5p inhibitor, and miR-NC were purchased from RiboBio Co., Ltd. (Guangzhou, China). The ICAM1 overexpression plasmid (pcDNA3.1-ICAM1) and empty vector pcDNA3.1 were constructed and verified by our laboratory. The dual-luciferase reporter assay kit was purchased from Promega (USA), and the CCK-8 and apoptosis detection kit were purchased from Beyotime Biotechnology (Shanghai, China).

### Preparation of CSE

CSE was prepared according to the method reported in previous studies^22^, to simulate cigarette smoke-induced airway epithelial cell injury *in vitro*. Briefly, smoke from 3 unfiltered commercial cigarettes was bubbled into 10 mL serum-free DMEM medium. The mixture was adjusted to pH 7.4 with 1 mol/L HCl, then filtered through a 0.22 μm sterile filter to remove large particles and bacteria. This solution was defined as 100% CSE stock solution, which was freshly prepared before each experiment and used within 30 min after dilution to ensure biological activity. The final working concentration of CSE for cell treatment was 5%, which was determined by pre-experiments.

### Cell transfection and grouping

BEAS-2B cells in the logarithmic growth phase were seeded into culture plates and cultured overnight until the confluence reached 60–70%. Cell transfection was performed using Lipofectamine 3000 reagent (Invitrogen, USA) according to the manufacturer’s instructions. After 6 h of transfection, the medium was replaced with complete medium containing 5% CSE for 24 h of treatment, and then the cells were collected for subsequent experiments. The cells were divided into 6 groups with clear treatment protocols as follows:

- Control group: BEAS-2B cells were routinely cultured without any transfection or CSE treatment;
- CSE injury group: BEAS-2B cells were treated with 5% CSE for 24 h without transfection;
- CSE + miR-NC group: BEAS-2B cells were transfected with miR-NC for 6 h, then treated with 5% CSE for 24 h;
- CSE + miR-129-5p mimic group: BEAS-2B cells were transfected with miR-129-5p mimic for 6 h, then treated with 5% CSE for 24 h;
- CSE + miR-129-5p inhibitor group: BEAS-2B cells were transfected with miR-129-5p inhibitor for 6 h, then treated with 5% CSE for 24 h; and
- Rescue group: BEAS-2B cells were co-transfected with miR-129-5p mimic and pcDNA3.1-ICAM1 overexpression plasmid for 6 h, then treated with 5% CSE for 24 h.

All subsequent cell functional experiments in this study were performed with 3 independent biological replicates for each group.

### Cell viability detection

Cell viability was evaluated using the Cell Counting Kit-8 (CCK-8) assay. BEAS-2B cells were seeded into 96-well plates at a density of 5×10^3^ cells/well, with 5 replicate wells set for each group. After the corresponding treatment, 10 μL of CCK-8 solution was added to each well, and the cells were incubated at 37^o^C for 4 h in the dark. The absorbance at 450 nm was detected by a microplate reader, and the relative cell viability of each group was calculated with the control group as 100%.

### Cell apoptosis detection

Cell apoptosis was detected using the Annexin V-FITC/PI apoptosis detection kit. After the corresponding treatment, BEAS-2B cells were digested with trypsin without EDTA, collected, and washed twice with pre-cooled PBS. The cells were resuspended in 100 μL of binding buffer, then stained with 5 μL of PI and 10 μL of Annexin V-FITC for 15 min at room temperature in the dark. Finally, 400 μL of binding buffer was added, and the apoptosis rate was detected by flow cytometry within 1 h.

### Dual-luciferase reporter assay

The ENCORI database was used to predict the potential binding site between miR-129-5p and the 3’UTR of ICAM1 mRNA. The wild-type (WT-ICAM1) and mutant-type (MUT-ICAM1) luciferase reporter vectors of ICAM1 3’UTR were constructed. HEK293T cells were co-transfected with the WT-ICAM1/MUT-ICAM1 vector and miR-129-5p mimic/miR-NC, respectively. After 48 h of transfection, the firefly luciferase activity and Renilla luciferase activity were detected using the Dual-Luciferase Reporter Assay System (Promega, USA). The relative luciferase activity was calculated by normalizing the firefly luciferase activity to the Renilla luciferase activity.

### Detection of inflammatory factors in cell supernatant

After the corresponding treatment, the cell culture supernatants from each group were collected. The levels of TNF-α and IL-6 in the supernatant were detected using ELISA kits, strictly following the manufacturer’s instructions.

### Statistical analysis

For the clinical cross-sectional study, the Shapiro-Wilk test was used for normality verification. Continuous data are presented as mean ± SD (normal distribution) or median (IQR) (non-normal distribution), and categorical data as frequency and percentage. Between-group comparisons adopted t-test/Mann-Whitney U test (two groups), ANOVA/Kruskal-Wallis H test with matched *post hoc* tests (multi-groups) for continuous variables, and chi-squared/Fisher’s exact test for categorical variables. Pearson/Spearman correlation, ROC curve analysis, and confounder-adjusted univariate/multivariate logistic regression were applied for correlation, diagnostic performance, and risk factor assessments, respectively.

For *in vitro* experiments (3 biological and 3 technical replicates), data are shown as mean ± SD. Two-group comparisons used an independent t-test, and multi-group comparisons adopted one-way ANOVA with Tukey’s/Dunnett T3 *post hoc* test.

All statistical analyses were performed using SPSS 27.0 (IBM, USA), and data visualization was conducted with GraphPad Prism 10.0 (GraphPad Software, USA). A two-sided p<0.05 was set as the threshold for statistical significance.

## RESULTS

### Baseline characteristics of the study subjects

A total of 126 COPD patients and 120 healthy controls were enrolled in this cross-sectional study. The baseline characteristics of all participants are shown in Table 1. There were no statistically significant differences between the COPD group and the control group in terms of age, gender distribution, and BMI. There were statistically significant differences between the two groups in smoking rate, pulmonary function indices (FEV_1_ % predicted, FEV_1_/FVC), and serum levels of inflammatory factors (TNF-α, IL-6).

**Table 1:** Baseline characteristics of participants in the cross-sectional study conducted at Hanyang Hospital affiliated to Wuhan University of Science and Technology, March 2022 – May 2024 (N=246)

*Characteristics*	*Healthy* *(N=120)* *Mean ± SD*	*COPD* *(N=126)* *Mean ± SD*	*p*
**Gender,** n (%)			0.163
Male	63 (52.50)	75 (59.52)	
Female	57 (47.50)	51 (40.48)	
**Age** (years)	64.89 ± 4.92	65.26 ± 4.38	0.524
**BMI** (kg/m^2^)	23.21 ± 2.58	23.10 ± 3.53	0.794
**Smoking,** n (%)	37 (30.83)	62 (49.21)	0.002
**Measurements**			
FEV_1_ (predicted, %)	95.14 ± 1.40	66.35 ± 1.69	<0.001
FEV_1_/FVC (%)	82.86 ± 0.89	61.06 ± 0.23	<0.001
TNF-α (pg/mL)	9.75 ± 0.48	26.54 ± 1.50	<0.001
IL-6 (pg/mL)	7.02 ± 1.24	16.05 ± 0.76	<0.001
**COPD grades,** n (%)			
I		25 (19.8)	
II		82 (65.1)	
III		19 (15.1)	

BMI: body mass index. COPD: chronic obstructive pulmonary disease. FEV_1_₁: forced expiratory volume in the first second. FVC: forced vital capacity. TNF-α: tumor necrosis factor-α. IL-6: interleukin-6. GOLD: Global Initiative for Chronic Obstructive Lung Disease.

### Expression of miR-129-5p and ICAM1 in the serum of COPD patients and healthy controls

**Figure 1:**
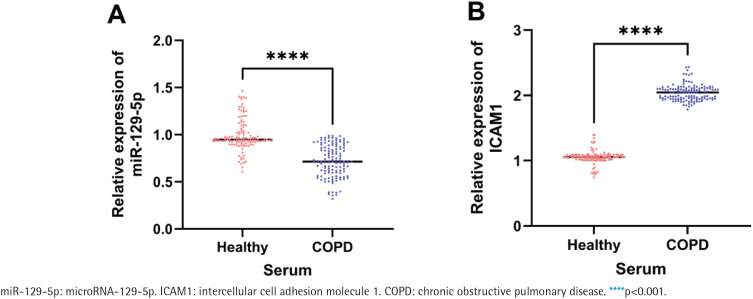
Relative expression of miR-129-5p and ICAM1 in serum from 120 healthy controls and 126 COPD patients: A) Relative expression level of miR-129-5p in serum from healthy controls and COPD patients; B) Relative expression level of ICAM1 in serum from healthy controls and COPD patients. The horizontal line represents the median of the relative expression level

qRT-PCR results showed that the relative expression level of serum miR-129-5p in the COPD group was significantly lower than that in the control group (p<0.001, Figure 1A), while the relative expression level of serum ICAM1 in the COPD group was significantly higher than that in the control group (p<0.001, Figure 1B).

### Association between miR-129-5p expression and COPD susceptibility

**Figure 2:**
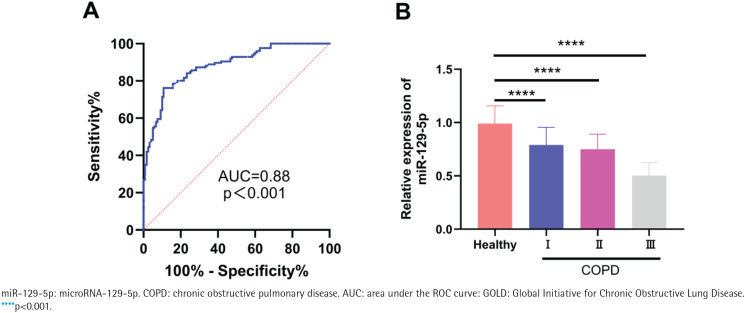
Evaluation of the association between serum miR-129-5p and COPD: A) Receiver operating characteristic (ROC) curve of serum miR-129-5p for distinguishing COPD patients from healthy controls; B) Relative expression level of serum miR-129-5p in healthy controls and COPD patients with different GOLD grades; the thin line above the bar represents the standard deviation (SD)

ROC curve analysis showed that serum miR-129-5p could distinguish COPD patients from healthy controls, with an AUC of 0.88 (95% CI: 0.84–0.92, p<0.001) (Figure 2A). The relative expression level of serum miR-129-5p decreased gradually with the increase of COPD severity grade (p<0.001, Figure 2B).

Univariate logistic regression analysis showed that miR-129-5p expression, smoking status, FEV_1_ % predicted, FEV_1_/FVC, TNF-α, and IL-6 were associated with COPD susceptibility (all p<0.05). Multivariate logistic regression analysis adjusted for gender, age, and BMI showed that low expression of miR-129-5p was an independent factor associated with COPD status (AOR=0.15; 95% CI: 0.08–0.26, p<0.001) (Table 2).

**Table 2:** Multivariate logistic regression analysis of factors associated with COPD susceptibility in the cross-sectional study (N=246)

*Variables*	*AOR*	*95% CI*	*p*
miR-129-5p (relative expression)	0.15	0.08–0.26	<0.001
Gender (male vs female)	1.88	0.96–3.65	0.065
Age (years) (per 1-year increase)	1.60	0.86–2.98	0.138
BMI (kg/m^2^) (per 1 kg/m² increase)	1.57	0.82–3.03	0.176
Current smoking (yes vs no)	2.53	1.37–4.69	0.003
FEV_1_ (per 1% predicted increase)	0.11	0.06–0.21	<0.001
FEV_1_/FVC (per 1% increase)	0.09	0.05–0.17	<0.001
TNF-α (pg/mL) (per 1 pg/mL increase)	2.59	1.38–4.85	0.003
IL-6 (pg/mL) (per 1 pg/mL increase)	3.04	1.65–5.63	<0.001

AOR: adjusted odds ratio. Model was adjusted for gender, age, and BMI. AOR values for continuous variables represent the change in odds of COPD per unit increase in the variable. Current smoking is defined as smoking at least 1 cigarette per day for more than 6 months; the percentage in Table 1 represents the proportion of current smokers in each group, and the AOR value reflects the association between current smoking status and COPD susceptibility. BMI: body mass index. COPD: chronic obstructive pulmonary disease. FEV₁_1_: forced expiratory volume in the first second. FVC: forced vital capacity. TNF-α: tumor necrosis factor-α. IL-6, interleukin-6.

### Correlation between miR-129-5p expression and inflammatory factors as well as pulmonary function in COPD patients

**Figure 3:**
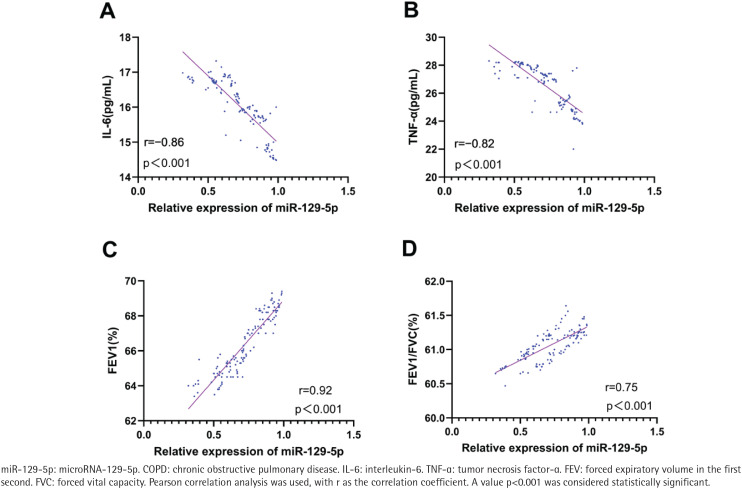
Correlation between serum miR-129-5p expression and inflammatory factors as well as pulmonary function indices in 126 COPD patients: A) Correlation between miR-129-5p expression and serum IL-6 level; B) Correlation between miR-129-5p expression and serum TNF-α level; C) Correlation between miR-129-5p expression and FEV_1_ % predicted; and D) Correlation between miR-129-5p expression and FEV_1_/FVC

Pearson correlation analysis showed that the relative expression level of serum miR-129-5p in COPD patients was negatively correlated with serum IL-6 level (r= -0.86; 95% CI: -0.90 – -0.81, p<0.001) ( Figure 3A) and TNF-α level (r= -0.82; 95% CI: -0.87 – -0.75, p<0.001) (Figure 3B), and was positively correlated with FEV_1_ % predicted (r=0.92; 95% CI: 0.89–0.94, p<0.001) (Figure 3C) and FEV_1_/FVC (r=0.75; 95% CI: 0.67–0.81, p<0.001) (Figure 3D).

### Verification of the targeting relationship between miR-129-5p and ICAM1

The ENCORI database predicted that there was a potential complementary binding site between miR-129-5p and the 3’UTR of ICAM1 mRNA (Supplementary file Figure 1A). Dual-luciferase reporter assay results showed that miR-129-5p mimic significantly inhibited the relative luciferase activity of WT-ICAM1 vector, but had no significant effect on the relative luciferase activity of MUT-ICAM1 vector (Supplementary file Figure 1B), confirming that miR-129-5p could specifically target and bind to the 3’UTR of ICAM1 mRNA.

### Effect of miR-129-5p on CSE-induced BEAS-2B cell injury

qRT-PCR results showed that compared with the control group, the relative expression level of miR-129-5p in BEAS-2B cells in the CSE injury group was significantly downregulated (p<0.001) (Supplementary file Figure 1C). Compared with the CSE + miR-NC group, the expression level of miR-129-5p in the CSE + miR-129-5p mimic group was significantly upregulated, and that in the CSE + miR-129-5p inhibitor group was significantly downregulated (p<0.001) (Supplementary file Figure 1C).

CCK-8 assay results showed that compared with the control group, the relative cell viability of the CSE injury group was significantly decreased (p<0.001) (Supplementary file Figure 1E). Compared with the CSE + miR-NC group, the relative cell viability of the CSE + miR-129-5p mimic group was significantly increased (p<0.001), and that of the CSE + miR-129-5p inhibitor group was significantly decreased (p<0.001) (Supplementary file Figure 1E).

Flow cytometry results showed that compared with the control group, the apoptosis rate of BEAS-2B cells in the CSE injury group was significantly increased (p<0.001) (Supplementary file Figure 1F). Compared with the CSE + miR-NC group, the apoptosis rate of the CSE + miR-129-5p mimic group was significantly decreased (p<0.001), and that of the CSE + miR-129-5p inhibitor group was significantly increased (p<0.001) (Supplementary file Figure 1F).

ELISA results showed that compared with the control group, the levels of TNF-α and IL-6 in the cell supernatant of the CSE injury group were significantly increased (p<0.001) (Supplementary file Figures 1G and 1H). Compared with the CSE + miR-NC group, the levels of TNF-α and IL-6 in the CSE + miR-129-5p mimic group were significantly decreased (p<0.001), and those in the CSE + miR-129-5p inhibitor group were significantly increased (p<0.001) (Supplementary file Figures 1G and 1H).

### Rescue experiment of ICAM1 on the protective effect of miR-129-5p

qRT-PCR results showed that compared with the CSE + miR-NC group, the expression level of ICAM1 in the CSE + miR-129-5p mimic group was significantly downregulated (p<0.001) (Supplementary file Figure 1D). Compared with the CSE + miR-129-5p mimic group, the expression level of ICAM1 in the rescue group was significantly upregulated (p<0.001) (Supplementary file Figure 1D).

Compared with the CSE + miR-129-5p mimic group, the relative cell viability of the rescue group was significantly decreased (p<0.001) (Supplementary file Figure 1E), the apoptosis rate was significantly increased (p<0.001) (Supplementary file Figure 1F), and the levels of TNF-α and IL-6 in the cell supernatant were significantly increased (p<0.001) (Supplementary file Figures 1G and 1H).

In addition, qRT-PCR results showed that the expression level of RELA in the CSE injury group was significantly higher than that in the control group (p<0.001) (Supplementary file Figure 1I). miR-129-5p overexpression significantly downregulated RELA expression in CSE-treated BEAS-2B cells (p<0.001), while ICAM1 overexpression reversed the inhibitory effect of miR-129-5p on RELA expression (p<0.001) (Supplementary file Figure 1I). The serum RELA level in COPD patients was significantly higher than that in healthy controls (p<0.001) (Supplementary file Figure 1J).

## DISCUSSION

This study systematically explored the association between miR-129-5p and COPD, as well as its regulatory role in CSE-induced bronchial epithelial cell injury. The core findings of this study are as follows: 1) serum miR-129-5p was significantly downregulated in COPD patients, and low expression of miR-129-5p was an independent factor associated with COPD status; 2) miR-129-5p expression was closely associated with inflammatory levels and pulmonary function impairment in COPD patients; and 3) miR-129-5p alleviated CSE-induced bronchial epithelial cell injury, inhibited inflammatory response, and reduced cell apoptosis by specifically targeting ICAM1 and downregulating RELA expression.

In recent years, the role of miRNAs in COPD has attracted considerable attention. Their abnormal expression suggests their involvement in disease occurrence and progression, making them promising potential therapeutic targets^23^. It has been confirmed that the expression levels of multiple miRNAs are dysregulated in COPD patients, such as significantly reduced miR-223 and miR-483 in lung tissues, and downregulated miR-335-5p in lung fibroblasts of smokers^13,24,25^. Notably, miR-129-5p has been well documented to exert broad anti-inflammatory activity in multiple disease models^16^, and its downregulation has been widely reported in various diseases, including primary lung cancer, where its expression is significantly reduced in tumor tissues and cell lines^19,20^. Consistent with these previous findings, our study found that serum miR-129-5p was significantly downregulated in COPD patients, and its expression level decreased gradually with the increase of COPD severity. ROC curve analysis showed that miR-129-5p had a good ability to distinguish COPD patients from healthy controls in this study, and multivariate logistic regression analysis confirmed that low expression of miR-129-5p was an independent factor associated with COPD susceptibility. These findings not only echo the previously reported expression pattern and biological function of miR-129-5p in inflammatory diseases and pulmonary disorders, but also, for the first time, reveal its potential as a circulating biomarker for COPD, laying a preliminary experimental foundation for subsequent exploration of COPD-related biomarkers, and further validation in large-scale, multi-center prospective clinical studies is warranted before any clinical application.

Bronchial epithelial cells are the core of the airway barrier, and their functional impairment is an early pathological event in COPD. CSE is a classic tool to simulate cigarette exposure-induced airway injury^26^ and BEAS-2B is an immortalized cell line with functional characteristics of normal human bronchial epithelial cells^27^. Therefore, this study established a CSE-induced BEAS-2B cell injury model to verify the biological function of miR-129-5p in airway epithelial injury. The results showed that CSE treatment significantly downregulated miR-129-5p expression in BEAS-2B cells, which was consistent with the expression trend in the serum of COPD patients. Overexpression of miR-129-5p significantly reversed CSE-induced decrease in cell viability, increase in apoptosis, and excessive release of inflammatory factors, while knockdown of miR-129-5p exacerbated the above cell injury. These *in vitro* experimental results are consistent with the clinical findings and confirm that miR-129-5p exerts a protective effect on CSE-induced bronchial epithelial cell injury and its downregulation may be involved in the occurrence and progression of COPD.

Chronic airway inflammation is the core pathological mechanism of COPD progression, and the NF-κB signaling pathway is a key pathway regulating the inflammatory response in COPD^28^. ICAM1 is an important adhesion molecule that mediates the adhesion and infiltration of inflammatory cells^29^ and is a downstream target gene of the NF-κB pathway^30^. Previous studies have confirmed that ICAM1 is highly expressed in the airway epithelium of COPD patients^31^ and mediates the infiltration of inflammatory cells and the release of pro-inflammatory factors^32^. RELA (p65) is the core functional subunit of NF-κB, and the activation of the NF-κB pathway can promote the transcription of pro-inflammatory factors such as TNF-α and IL-6, forming a positive feedback loop of inflammatory response^33-35^. In this study, a dual-luciferase reporter assay confirmed that miR-129-5p could specifically target and bind to the 3’UTR of ICAM1 mRNA, and negatively regulate the expression of ICAM1. Overexpression of miR-129-5p significantly downregulated the expression of ICAM1 and RELA in CSE-treated BEAS-2B cells, inhibited the release of inflammatory factors, and alleviated cell injury. Rescue experiments showed that overexpression of ICAM1 could abrogate the protective effect of miR-129-5p on bronchial epithelial cells. These results are highly consistent with previous reports that miR-129-5p alleviates inflammatory injury by negatively regulating the NF-κB signaling pathway in LPS-induced acute kidney injury^17^, and that it can directly target inflammatory factors to suppress the inflammatory cascade^19^. Taken together, our findings suggest that miR-129-5p alleviates CSE-induced airway epithelial cell injury and inflammatory response by targeting ICAM1 and inhibiting the activation of the NF-κB/RELA signaling pathway, which reveals the potential molecular mechanism of miR-129-5p in regulating COPD progression and further expands the functional landscape of miR-129-5p in chronic inflammatory diseases.

### Limitations

Several limitations of this study should be noted. The single-center cross-sectional design with small sample size may bring residual confounding, requiring large multi-center prospective validation. Functional experiments were limited to *in vitro* cell models, lacking *in vivo* animal verification. Only mRNA-level regulation of ICAM1 and RELA was verified, without protein-level validation. Only the miR-129-5p/ICAM1/NF-κB axis was explored, with other potential targets of miR-129-5p in COPD unclarified.

## CONCLUSIONS

Downregulation of serum miR-129-5p was associated with COPD status. miR-129-5p was noted to alleviate CSE-induced human bronchial epithelial cell injury by targeting and inhibiting ICAM1 expression and downregulating the RELA signaling pathway. This study provides preliminary experimental evidence for elucidating the pathogenesis of COPD, and further large-scale, multi-center studies and *in vivo* experiments are needed to verify the clinical application value of miR-129-5p.

## Supplementary Material



## Data Availability

The data supporting this research are available from the authors on reasonable request.
