# Supplementary material for: MiR-129-5p is associated with chronic obstructive pulmonary disease status and alleviates cigarette smoke extract-induced human bronchial epithelial cell injury
Source: Tob Induc Dis. 2026 Jun 30;24:10.18332/tid/221191. doi: 10.18332/tid/221191 (PMC13320935; doi:10.18332/tid/221191)
Supplement: Supplementary file 1 [file TID-24-101-s1.pdf]

Name	Sequence (5'→3')
miR-129-5p-forward	CGGCGGTGAGGTGTTTGG
miR-129-5p-reverse	GTGCAGGGTCCGAGGT
U6-forward	CTCGCTTCGGCAGCACA
U6-reverse	AACGCTTCACGAATTTGCGT
GAPDH-forward	ATCACCATCTTCCAGGAGCGA
GAPDH-reverse	CCTTCTCCATGGTGGTGAAGAC
ICAM1-forward	CAGCCAGGTGGTGATGTTTC
ICAM1-reverse	GTGGCTGTCTGTCTTGGTTG
RELA-forward	GAAGCCCTGAGGGAAGAAAT
RELA-reverse	CAGGGCTGCTTTTGCTTATC

Supplementary Table 1. Primer sequences for quantitative real-time polymerase chain reaction (qRT-PCR) in this study

Note: All qRT-PCR reactions were performed on the Applied Biosystems 7500 Real-Time PCR System with a two-step amplification procedure; the relative expression of target genes was calculated using the $2^{-\Delta\Delta Ct}$ method.

Abbreviations: qRT-PCR, quantitative real-time polymerase chain reaction; miR-129-5p, microRNA-129-5p; ICAM1, intercellular cell adhesion molecule 1; GAPDH, glyceraldehyde-3-phosphate dehydrogenase.

Supplementary Figure 1. Verification of the targeting relationship between miR-129-5p and ICAM1, and the functional experiment results in CSE-induced BEAS-2B cells (A) Predicted complementary binding site between miR-129-5p and the 3'UTR of ICAM1 mRNA; (B) Dual-luciferase reporter assay verified the targeted binding between miR-129-5p and ICAM1; (C) Relative expression of miR-129-5p in BEAS-2B cells of each group; (D) Relative expression of ICAM1 in BEAS-2B cells of each group; (E) Relative cell viability of BEAS-2B cells in each group detected by CCK-8 assay; (F) Apoptosis rate of BEAS-2B cells in each group detected by flow cytometry; (G-H) Levels of IL-6 and TNF-α in the cell supernatant of each group detected by ELISA; (I) Relative expression of RELA in BEAS-2B cells of each group; (J) Relative expression of RELA in serum from healthy controls and COPD patients.

Abbreviations: miR-129-5p, microRNA-129-5p; ICAM1, intercellular cell adhesion molecule 1; CSE, cigarette smoke extract; 3'UTR, 3' untranslated region; WT, wild-type; MUT, mutant-type; miR-NC, negative control microRNA; CCK-8, Cell Counting

© 2026 Liu D. et al.
DOI: 10.18332/tid/221191

Kit-8; ELISA, enzyme-linked immunosorbent assay; IL-6, interleukin-6; TNF-α, tumor necrosis factor-α; COPD, chronic obstructive pulmonary disease.

Note: The thin line above the bar represents the standard deviation (SD); ****P < 0.001

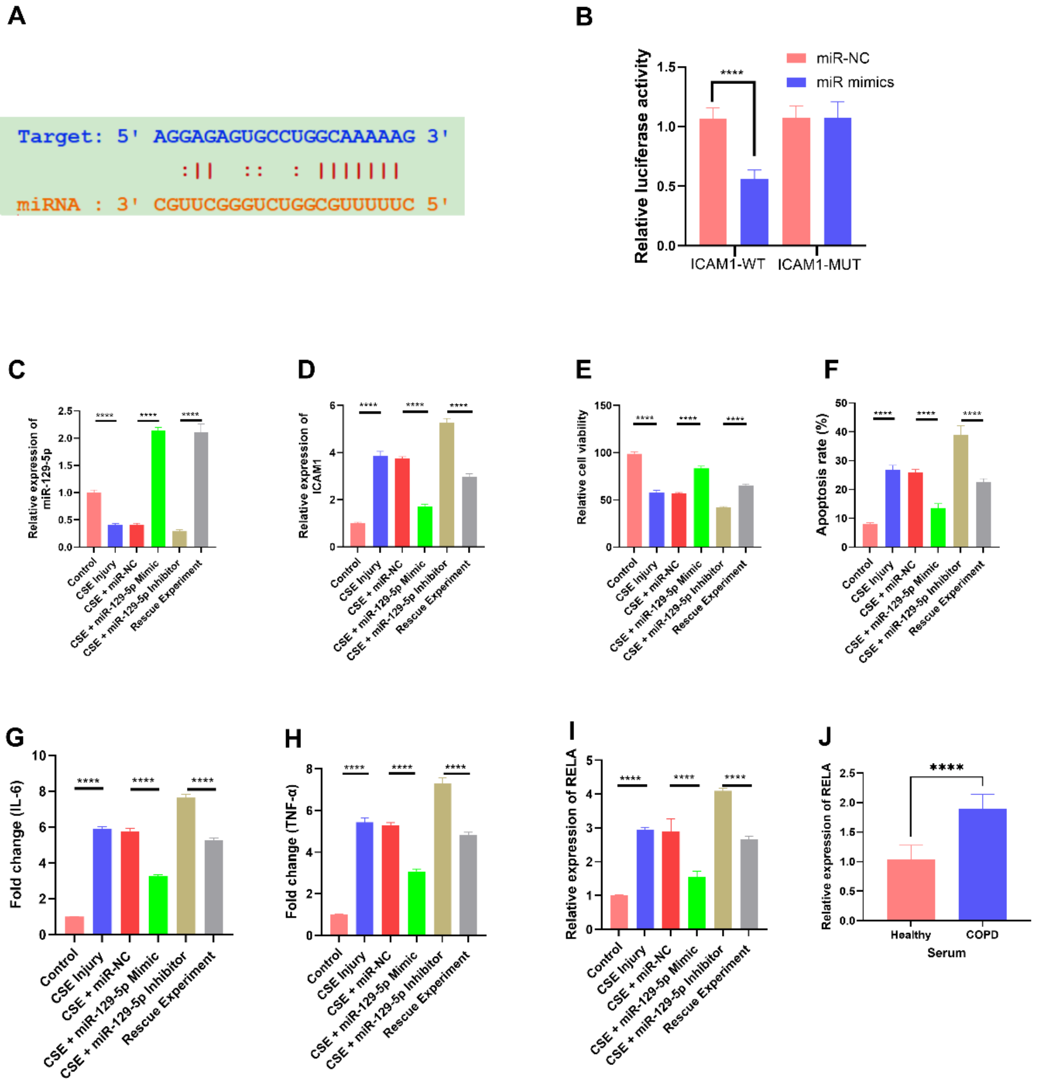

This content has been provided by the author(s) and has not been reviewed, verified, or endorsed by European Publishing. It may not have undergone peer review. The views, opinions, and recommendations expressed are solely those of the author(s) and do not necessarily reflect the position of European Publishing. European Publishing accepts no responsibility or liability for any consequences arising from the use of, or reliance on, this content.

© 2026 Liu D. et al.
DOI: 10.18332/tid/221191
